# Affibody PET Imaging of HER2-Expressing Cancers as a Key to Guide HER2-Targeted Therapy

**DOI:** 10.3390/biomedicines12051088

**Published:** 2024-05-14

**Authors:** Nina Eissler, Renske Altena, Ali Alhuseinalkhudhur, Olga Bragina, Joachim Feldwisch, Guido Wuerth, Annika Loftenius, Nikolai Brun, Rimma Axelsson, Vladimir Tolmachev, Jens Sörensen, Fredrik Y. Frejd

**Affiliations:** 1 Affibody AB, 17165 Solna, Sweden; 2 Department of Oncology-Pathology, Karolinska Institutet, 17164 Solna, Sweden; 3 Medical Unit Breast, Endocrine Tumors and Sarcoma, Theme Cancer, Karolinska Comprehensive Cancer Center, Karolinska University Hospital, 17164 Solna, Sweden; 4 Medical Radiation Physics and Nuclear Medicine, Functional Unit of Nuclear Medicine, Karolinska University Hospital, 14157 Huddinge, Sweden; 5 Nuclear Medicine and PET, Department of Surgical Sciences, Uppsala University, 75310 Uppsala, Sweden; 6 Department of Immunology, Genetics and Pathology, Uppsala University, 75310 Uppsala, Sweden; 7 Department of Nuclear Therapy and Diagnostic, Cancer Research Institute, Tomsk National Research Medical Center, Russian Academy of Sciences, 634055 Tomsk, Russia; 8 Research Centrum for Oncotheranostics, Research School of Chemistry and Applied Biomedical Sciences, Tomsk Polytechnic University, 634050 Tomsk, Russia; 9 Department of Molecular Medicine and Surgery, Karolinska Institutet, 14152 Stockholm, Sweden

**Keywords:** HER2, breast cancer, diagnostics, molecular imaging, Affibody molecules, clinical trials

## Abstract

Human epidermal growth factor receptor 2 (HER2) is a major prognostic and predictive marker overexpressed in 15–20% of breast cancers. The diagnostic reference standard for selecting patients for HER2-targeted therapy is based on the analysis of tumor biopsies. Previously patients were defined as HER2-positive or -negative; however, with the approval of novel treatment options, specifically the antibody–drug conjugate trastuzumab deruxtecan, many breast cancer patients with tumors expressing low levels of HER2 have become eligible for HER2-targeted therapy. Such patients will need to be reliably identified by suitable diagnostic methods. Biopsy-based diagnostics are invasive, and repeat biopsies are not always feasible. They cannot visualize the heterogeneity of HER2 expression, leading to a substantial number of misdiagnosed patients. An alternative and highly accurate diagnostic method is molecular imaging with radiotracers. In the case of HER2, various studies demonstrate the clinical utility and feasibility of such approaches. Radiotracers based on Affibody^®^ molecules, small, engineered affinity proteins with a size of ~6.5 kDa, are clinically validated molecules with favorable characteristics for imaging. In this article, we summarize the HER2-targeted therapeutic landscape, describe our experience with imaging diagnostics for HER2, and review the currently available clinical data on HER2-Affibody-based molecular imaging as a novel diagnostic tool in breast cancer and beyond.

## 1. Introduction

Human epidermal growth factor receptor 2 (HER2) is overexpressed and/or amplified in around 15–20% of breast cancer patients and expressed to a lesser degree in a large fraction of the remaining breast cancers [1,2,3]. Many other solid tumors also show high frequencies of HER2 expression, e.g., gastric and gastroesophageal carcinoma, ovarian cancer, pancreatic cancer, and colorectal cancer [4]. The use of HER2-targeted therapies has dramatically improved the outcome and survival of HER2-expressing breast cancer patients over the last decades since the approval of the first HER2-targeted antibody, trastuzumab, in 1998 [5]. To date, four tyrosine kinase inhibitors (pyrotinib with approval in China only), three monoclonal antibodies, and two antibody–drug conjugates (ADC) directed against HER2 have been approved and implemented in clinical practice. These therapies are used primarily for breast cancer but also for gastric cancer and non-small cell lung cancers (NSCLC) with activating HER2 mutations (Table 1) [6]. Furthermore, several trastuzumab biosimilars, as well as subcutaneous formulations of trastuzumab and pertuzumab, have entered the market. Multiple clinical trials are ongoing in breast cancer and other HER2-expressing cancers, evaluating promising novel drug formats, including (combinations of) HER2-targeted bispecific antibodies, ADCs, radiotherapies, and cancer vaccines [7,8]. Notably, the novel ADC trastuzumab deruxtecan has shown efficacy in patients with low HER2 expression in the DESTINY-Breast04 trial [9], thereby opening a new treatment opportunity for a large patient group previously not eligible for HER2-targeted therapies. Trastuzumab deruxtecan demonstrated efficacy in a basket trial investigating therapeutic outcomes in a tissue-agnostic manner across different HER2-positive indications [10].

The detection of HER2 expression and, consequently, the decision on whether a patient will receive HER2-targeted therapy is routinely assessed by immunohistochemistry (IHC) and/or in situ hybridization (ISH) assays performed on a biopsy from a primary tumor or metastasis. Thus, HER2-positivity has previously been defined as IHC3+ and IHC2+/ISH+. Whereas HER2-negativity has been defined as IHC2+/ISH−, IHC1+, and IHC0, new research and clinical data suggest that the distinction between HER2-low and true HER2-negative (IHC0) will become more important with the implementation of novel therapeutics in clinical practice [11]. Heterogeneity of HER2 expression in cancer has been recognized as a major challenge, and a plethora of studies have demonstrated the spatial and temporal heterogeneity of HER2 expression and its impact on treatment outcomes [3,12,13,14,15]. Although these limitations have been a topic of debate for over 20 years, no alternative diagnostic methods have been implemented in clinical routines to guide treatment decisions for HER2-targeting agents. In clinical practice, patients may not be offered HER2-directed therapies because of false-negative biopsy results. Conversely, patients whose tumors lose HER2 expression after initial treatment may remain on a therapy that has no or only limited benefit but bears the risk of unnecessary adverse events due to assumed HER2-positivity [14,16,17]. An analysis of more than 21,000 early-stage breast cancer patients in Japan revealed that out of 2811 patients whose tumors were HER2-positive before neoadjuvant chemotherapy, 601 patients (21.4%) had lost HER2 expression after therapy. In the same study, it was shown that 340 (3.4%) of the 9947 patients with tumors previously classified as HER2-negative had HER2-positive tumors following therapy [18]. In another clinical trial of 164 patients with early-stage breast cancer, tumor heterogeneity was assessed by comparing HER2 expression on biopsy tissue from two locations of the same tumor. Patients with intratumoral heterogeneity of HER2 expression showed no response to neoadjuvant therapy with trastuzumab emtansine plus pertuzumab, while those with HER2-positive tumors without heterogeneity showed a pathological complete response in 55% of cases [17]. These examples highlight the importance of implementing diagnostic tools that allow the assessment of HER2 status before, during, or after therapeutic interventions in all tumor lesions. A better understanding of the inter- and intratumoral heterogeneity of HER2 expression is crucial for making optimized and personalized treatment decisions to maximize patient benefit. Furthermore, there is an emerging need to better understand the impact of modern HER2-targeting drugs, such as trastuzumab and deruxtecan, on patient outcomes and particularly, their role in HER2-low disease. Robust methods that can overcome the shortcomings of biopsy-based determination of HER2 status will need to be developed to characterize HER2-low disease and enable visualization of HER2 heterogeneity [9,14,19]. 

## 2. Application of Diagnostic Radiopharmaceuticals in Oncology

Positron emission tomography (PET) and single-photon emission computed tomography (SPECT) are both imaging techniques that can detect the distribution of radiotracers in the tissues of the body. For PET, positron-emitting radionuclides such as fluorine-18 and gallium-68 are often used. When an emitted positron meets an electron, annihilation takes place, and consequently, two 511-keVgamma quanta are emitted. These are detected in a coincidence at 180 degrees apart from one another by the scintillation detectors of a PET scanner, allowing very precise localization of the tracer. From the signal detected by PET cameras, a 3D image can be reconstructed that visualizes the quantitative distribution of the radiotracer in the body. For SPECT imaging, on the other hand, gamma photons from radionuclides decaying in the tissue of the patient are detected. Radionuclides used for SPECT imaging include technetium-99m and indium-111. Gamma quanta are registered by a scintillation camera to create a 2D image. A computational analysis of multiple 2D images acquired at various angles results in a 3D image of the radiation distribution in the tissue. Each technique has its advantages and limitations, which are briefly described in Table 2 and reviewed in detail elsewhere [20,21].

Various diagnostic compounds for PET and SPECT imaging have been approved. A prominent tumor-agnostic imaging compound is [^18^F]F-2-fluoro-2-deoxy-D-glucose ([^18^F]FDG). [^18^F]FDG PET utilizes the abnormal glucose metabolism of tumors to allow visualization of areas with increased glucose uptake. In clinical practice [^18^F]FDG PET is combined with computed tomography (CT) and, as such, can provide a tool for staging or restaging tumors, detecting recurrence, and allowing monitoring of treatment responses in some tumor types [22,23,24]. Examples of other radiopharmaceuticals with non-specific accumulation in neoplastic malignancies include gallium-67 citrate, thallium-201 chloride, and technetium-99m sestamibi [25].

As opposed to the tumor-agnostic detection of metabolically active lesions by, e.g., [^18^F]FDG PET, many diagnostic radioligands specifically bind to and enable visualization of antigens or receptors expressed on tumor cells (see Table 3 for key diagnostic radioligands). Prominent examples are somatostatin receptor (SSTR)-targeted radioligands that visualize SSTR-positive neuroendocrine tumors. They are utilized to identify patients who will benefit from treatment with the radiotherapeutic drug lutetium Lu-177 dotatate, which is almost identical to the imaging agent but radiolabeled with the beta emitter lutetium-177. Similarly, PSMA-targeting radioligands help identify patients eligible for targeted radiotherapy with a second approved radioligand therapy, lutetium Lu-177 vipivotide tetraxetan. These diagnostics are part of a theranostic pair, in which the diagnostic imaging compound is closely related to a therapeutic radioligand and can therefore be utilized to stratify patients that will benefit most from such therapy [26]. 

Targeted radioligands can also be utilized to guide treatment decisions outside of the theranostic context. A prominent example is fluoroestradiol F-18 (FES PET) for the detection of estrogen receptor-positive breast cancer lesions by PET imaging as an adjunct to biopsy staging. Among breast cancer patients, around 70% have tumors expressing estrogen receptors, which is a major prognostic indicator [27,28]. As confirmed recently in the IMPACT trial, which included 200 patients with newly diagnosed metastatic breast cancer, a key advantage of FES PET imaging is its potential to non-invasively and with high sensitivity and specificity determine the estrogen receptor status throughout the body in patients with metastatic disease. [29]. Comparable imaging agents to identify HER2 expression in breast cancer are currently not approved and are not routinely used in clinical practice. However, various studies have highlighted the benefits of, for example, trastuzumab-based HER2-imaging agents [30,31,32,33]. 

The most advanced non-antibody imaging agent directed against HER2 belongs to the Affibody class of molecules. Affibody molecules are a class of small (~6.5 kDa) engineered affinity proteins that have been extensively investigated for imaging purposes [34,35], including imaging of both HER2 [36] and EGFR [37] expression. Within the Affibody drug class, high affinity binders to a large array of targets have been generated. Of note, the safety of this novel drug class has been demonstrated in more than 900 patients receiving different Affibody molecules. These include HER2 imaging tracers, which will be reviewed below, as well as a therapeutic Affibody molecule-blocking interleukin 17 (izokibep) that was recently demonstrated to be safe and efficacious in more than 750 patients with monthly high-dose administrations for up to three years [38]. In the following sections of this review, we provide an overview of the clinical experience in HER2 molecular imaging with a focus on Affibody-based HER2 radioligands and describe the applicability and utility of such ligands as novel diagnostic tools.

## 3. Assessing HER2 Status in Cancer Patients by Molecular Imaging

Molecular imaging with PET or SPECT for HER2 is a non-invasive diagnostic method that can be used to repeatedly assess whole-body HER2 status. Utilizing molecular imaging as a diagnostic tool can help overcome challenges and shortcomings associated with repeat biopsies and lesions not amenable to biopsy. Imaging also enables the detection of tumor heterogeneity. Various radiolabeled biomolecules for imaging of HER2 and other tumor targets have been tested as diagnostics in cancer patients, including radiolabeled trastuzumab and pertuzumab, nanobodies, antibody fragments, and other molecules, and are reviewed in detail elsewhere [39,40,41]. Small-scale clinical trials have shown a correlation between high uptake values of HER2-imaging tracers and response to HER2-targeted therapy. A pilot study including 10 patients with metastatic HER2-positive breast cancer utilized PET imaging with [^18^F]FDG PET and [^64^Cu]Cu-DOTA-trastuzumab before treatment with trastuzumab emtansine. Patients with higher tracer uptake had better response rates and a longer duration of response compared to patients with tracer uptake under a certain threshold [30]. These results corroborated the outcome of the earlier ZEPHIR trial. The final results of this trial, including 90 breast cancer patients, were recently published and show the high negative predictive value of [^89^Zr]Zr-trastuzumab PET alone or in combination with [^18^F]FDG PET in predicting lesion-based and patient-based responses to HER2-targeted therapy with trastuzumab emtansine (T-DM1) [31,42]. The outcome of this study clearly demonstrates that HER2 PET imaging can detect HER2-positive lesions in breast cancer patients and identify patients with a low probability of response to T-DM1.

To increase the accuracy of HER2 assessments during diagnostic workup, an imaging agent should enable high-contrast images to be acquired, ideally within a few hours after tracer injection [43]. Full-sized monoclonal antibodies (molecular weight ~150 kDa) are not ideal molecules due to their very long plasma half-lives, resulting in image acquisition days after tracer administration. Smaller binding molecules such as nanobodies (~14 kDa) can detect HER2 avid metastases in patients on the day of injection [44], but it has been argued that the ideal molecular size would be below 10 kDa to allow efficient extravasation and tumor penetration [45]. Affibody molecules (~6.5 kDa) are within this ideal molecular size range and have been demonstrated to be highly suitable tracers for PET and SPECT molecular imaging, enabling image acquisition within hours after tracer injection and generating high-contrast images. Considering the comprehensive clinical experience with Affibody molecules, and in particular with Affibody-based imaging tracers, they can be considered safe and efficacious tools for molecular imaging of cancer patients.

## 4. Clinical Experience of HER2-Affibody Molecular Imaging in HER2-Positive Breast Cancer Patients

**Figure 1:**
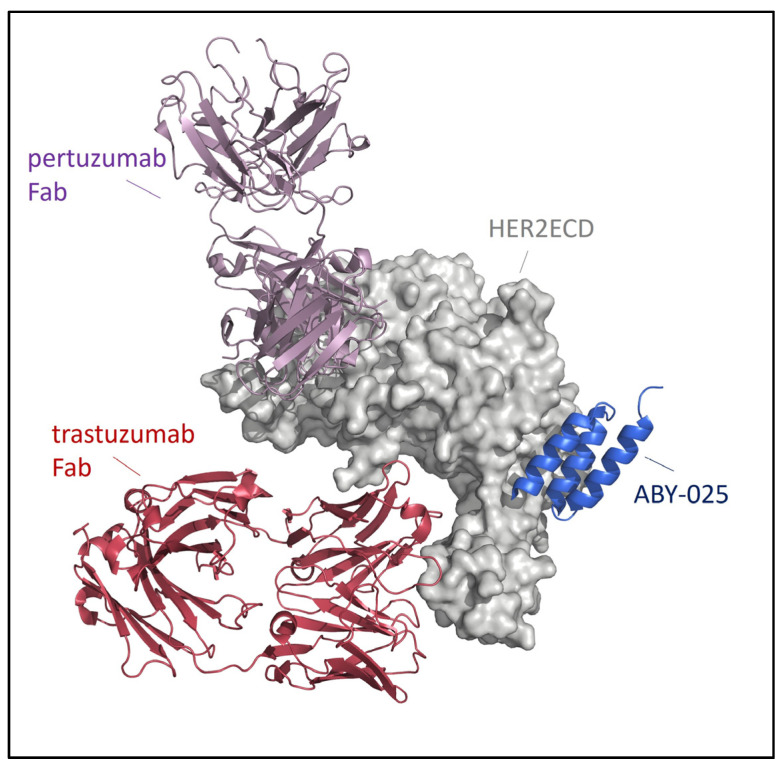
ABY-025 binds with picomolar affinity to the extracellular domain of HER2 (HER2ECD), an epitope distinct to the binding sites of therapeutic antibodies trastuzumab and pertuzumab. Fab: fragment antigen-binding region of a monoclonal antibody.

ABY-025 (tezatabep matraxetan) is a 7.55 kDa Affibody molecule binding specifically and with picomolar affinity to human HER2 [46]. Its utility for molecular SPECT or PET imaging of HER2 expression has been demonstrated in various preclinical and clinical studies. Notably, ABY-025 binds to a different epitope than the therapeutic antibodies trastuzumab and pertuzumab (see Figure 1), and imaging with this ligand is therefore not impaired by simultaneous HER2-targeted antibody therapy with currently approved agents [46,47].

The unique technical aspects of Affibody ligands as a drug class for molecular imaging are reviewed in detail elsewhere [35].

To date, a number of publications reporting a total of 188 breast cancer patients imaged with either the Affibody tracer ABY-025, ABY-025-derivatives, or closely related HER2-targeting Affibody molecules have been identified (Table 4). Furthermore, more than 30 patients with gastric or gastroesophageal carcinoma have undergone HER2-Affibody PET imaging, and additional studies in this patient group are ongoing (Table 5). Importantly, no safety issues or adverse events attributed to the imaging compound were reported in any of these studies, demonstrating a favorable benefit-risk profile of this imaging tracer. The first publication describing an Affibody imaging tracer with the Affibody ligand ABY-002, labeled with indium-111 or gallium-68 for SPECT or PET imaging, respectively, is, to our knowledge, also the first report on a non-immunoglobulin-based protein scaffold imaging tracer [48]. In this study, HER2-Affibody imaging was compared to previous [^18^F]FDG PET results in patients with breast cancer. Nine out of eleven [^18^F]FDG PET positive lesions could be detected by ABY-002. This first study highlighted the potential of the technology: HER2 positive metastases not amenable to biopsy could be identified, whole-body HER2 status could be visualized, and both quantitative and qualitative information not accessible through standard imaging procedures or biopsy analysis could be obtained. 

Following up on these promising first-in-human results, Sörensen et al. conducted several imaging trials in metastatic breast cancer patients, utilizing ABY-025 initially labeled with indium-111 for SPECT in seven patients and thereafter labeled with gallium-68 for PET. The trials covered a total of 63 ABY-025-imaged breast cancer patients from the same clinical center in Uppsala, Sweden [36,49,50]. In the first seven metastatic breast cancer patients (five HER2-positive and two HER2-negative patients according to previous biopsy), analyzed by SPECT imaging utilizing [^111^In]In-ABY-025, HER2-positive lesions showed clear tracer avidity, while uptake in HER2-negative lesions was low [49]. In all lesions that were subsequently verified by imaging-guided biopsy, HER2-IHC analyses correlated with either high or low contrast uptake for HER2-positive (defined as IHC3+ or FISH+; IHC2+ and FISH+) or negative (defined as IHC2+/ISH−, IHC1+, or IHC0) biopsy results, respectively. Notably, a brain metastasis not identified by [^18^F]FDG PET could be visualized with [^111^In]In-ABY-025 SPECT imaging in one patient, and this lesion was confirmed to be HER2-positive by IHC after surgical removal. No ABY-025-related adverse events were reported in this study, and furthermore, up to 6 weeks after tracer injection, no anti-drug antibodies could be detected. There was no correlation between tracer organ uptake and the release of serum HER2. Despite promising results, certain limitations were identified in this study: quantification of tracer uptake required dual imaging timepoints, and smaller metastases visualized by [^18^F]FDG PET could not be detected by HER2-SPECT imaging. These limitations may be due to the lower resolution of SPECT imaging in comparison to PET. Therefore, a follow-up trial was conducted to explore the clinical utility of [^68^Ga]Ga-ABY-025 PET imaging in 16 breast cancer patients, including both HER2-positive and HER2-negative patients as defined by biopsy [36]. After determining an optimized injected peptide mass in the first 10 patients, five additional patients were imaged twice with a one-week interval as a test–retest of uptake in individual lesions. A high correlation was shown between the two tests (r = 0.996). Here, [^68^Ga]Ga-ABY-025 PET imaging could discriminate between positive and negative lesions and showed a high correlation with corresponding biopsy results. The standardized uptake value (SUV) of lesions classified as HER2-positive by histology versus the SUV of HER2-negative lesions were significantly different in this study at both peptide doses tested and at all imaging time points. In the high peptide dose group, the mean SUVmax at the 4 h imaging time point was 5.5-fold higher in HER2-positive versus HER2-negative lesions, and there was a significant correlation between HER2-scoring by biopsy-based IHC and [^68^Ga]Ga-ABY-025 SUV. Importantly, treating physicians changed therapeutic intervention in three patients in this study based on ABY-025 PET results: two patients with tumors that were defined as HER2-negative by previous biopsies showed very high tracer uptake, and the lesions could be confirmed as HER2-positive by additional imaging-guided biopsy analyses. These patients were consequently treated with trastuzumab. A third patient had a primary HER2-positive tumor but showed very low ABY-025 avidity, and biopsies from two locations confirmed HER2-negative status. As a result, trastuzumab treatment was stopped for this patient. The results gained in this small patient cohort demonstrate the impact HER2-imaging could have if utilized broadly for optimizing patients’ therapy in clinical practice.

To understand if imaging with [^68^Ga]Ga-ABY-025 PET could predict early response to HER2-targeted therapy, Sörensen and colleagues conducted a phase II trial (NCT03655353). The interim results of this study were recently published [50] and report on 40 breast cancer patients with known HER2-positive status. Patients had either primary or metastatic breast cancer and were either treatment-naïve or had received up to three or more than three previous therapies.

**Figure 2:**
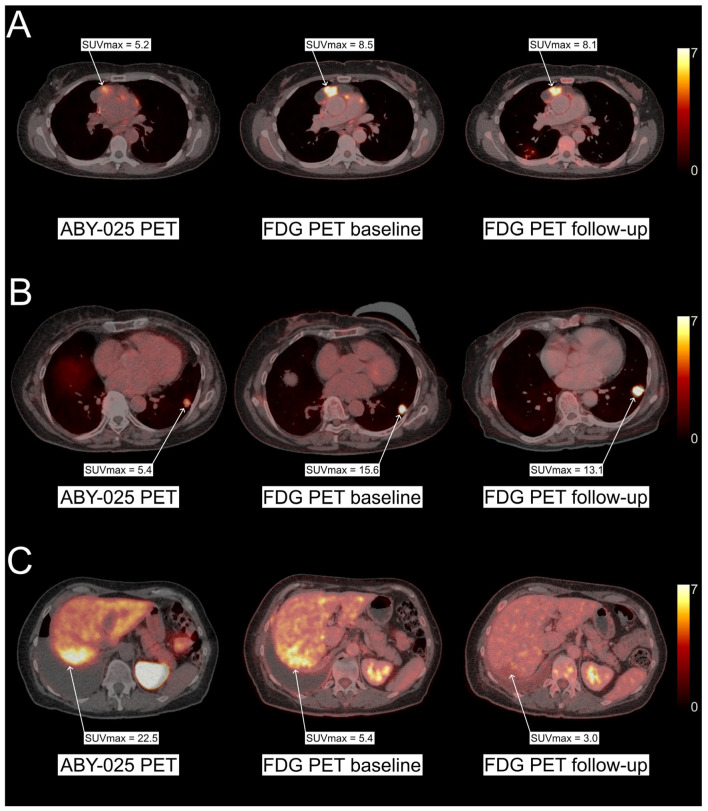
Representative images of PET scans conducted in the Affibody-3 trial (NCT03655353) provided by Jens Sörensen and colleagues. (**A**) (pt3): Breast cancer (BC) patient with biopsy-confirmed HER2-positive mediastinal metastasis (biopsy results: IHC2+/ISH+), [^68^Ga]Ga-ABY-025 uptake at baseline was low (SUVmax = 5.2). No metabolic response was observed after receiving two cycles of HER2-targeted therapy (**B**) (pt5): BC patient with biopsy confirmed HER2-positive lung metastasis (biopsy results: IHC3+/ISH−) with low [^68^Ga]Ga-ABY-025 uptake at baseline (SUVmax = 5.4). The patient had disease progression despite receiving three cycles of HER2-targeted therapy. (**C**) (pt15): BC patient with biopsy confirmed HER2-negative liver metastasis (biopsy results: IHC1+/ISH−). [^68^Ga]Ga-ABY-025 PET showed high uptake in tumor lesions (SUVmax = 22.5), and the patient showed a good metabolic response after two cycles of HER2-targeted therapy. Therapies received during the study period: Patient 3: epirubicin + cyclophosphamide + trastuzumab + pertuzumab; Patient 5: trastuzumab emtansine (FDG-PET after three cycles in this patient); Patient 15: docetaxel + trastuzumab + pertuzumab.

During the study, they were treated with two cycles ofHER2-targeted therapy (trastuzumab plus pertuzumab and chemotherapy in the neoadjuvant setting or at first recurrence, trastuzumab emtansine in the case of multiple recurrence). Early response was assessed by comparing the metabolic response measured by [18F]FDG PET at baseline and after two treatment cycles (see Figure 2). HER2-status was assessed by [68Ga]Ga-ABY-025 PET before the two treatment cycles, and one imaging-guided biopsy per patient was taken to reassess HER2 status by IHC/ISH. Strikingly, 12 out of 40 patients showed a mismatch in HER2-expression between biopsy and imaging when a cut-off SUVmax of 6.0 was applied. In the patient group with metastatic breast cancer, the same cutoff predicted early metabolic response with a sensitivity of 86% and specificity of 67% in soft tissue lesions, while HER2 status by biopsy showed no predictive value. A significant association between HER2 status by biopsy and [^68^Ga]Ga-ABY-025 uptake could not be established in this study. The authors concluded that the use of [^68^Ga]Ga-ABY-025 PET can be an adjunct diagnostic to predict the HER2 status required to induce early metabolic tumor remission after HER2-targeted therapy. Further studies are required to establish the clinical utility of ABY-025 PET imaging as a diagnostic tool for the detection of HER2 status and as a predictor of response to HER2-targeted therapy.

**Table 4:** Affibody-based HER2 molecular imaging clinical trials in breast cancer. BC: breast cancer; FiH: first in human.

Affibody Ligand	HER2 Status	Radioisotope (PET/SPECT)	Number of Patients	Patient Population	Study Design	References
ABY-002	pos	^111^In (SPECT); ^68^Ga (PET)	3	Recurrent metastatic BC	FiH, case studies	[48]
ABY-025	pos/neg	^111^In (SPECT)	7	Metastatic BC with a known HER2 classification of the primary tumor	Prospective, open-label, non-randomized FiH	[49,51]
ABY-025	pos/neg	^68^Ga (PET)	16	Metastatic BC with a known HER2 classification of the primary tumor	Prospective, open-label, non-randomized phase 1/2	[36,51,52]
NOTA-Mal-Cys-MZHer342	pos/neg	^68^Ga (PET)	2	BC	Case studies	[53]
HEHEHE-ZHER2-GGGC	pos	^99m^Tc (SPECT)	30	Suspected diagnosis of HER2-positive BC	Prospective, open label	[54]
NOTA-Mal-Cys-MZHer342	pos/neg	^68^Ga (PET)	24	Histopathologically confirmed BC, with unclear HER2 status	Prospective, open label	[55]
GE-226	pos	^18^F (PET)	20	Locally advanced or metastatic BC	Prospective, open label	[56]
RESCA-HER2-BCH/NOTA-HER2-BCH	pos/neg	^18^F (PET)	5	BC with histopathological results of HER2 status	Prospective, open label	[57]
ABY-025	pos	^68^Ga (PET)	40	Newly diagnosed stage II-III primary BC, planned for neoadjuvant therapy or confirmed progression in metastatic BC, and planned for anti-HER2 targeted therapy concomitant with chemotherapy	Prospective, open-label, non-randomized phase 2	[50]
ABY-025	low	^68^Ga (PET)	10	metastatic BC	Prospective, open-label, non-randomized phase 2	[58]
ZHER2:41071	pos/neg	^99m^Tc (SPECT)	31	Five HER2-positive and five HER2-low/negative BC patients per mass dose cohort (three cohorts, 500, 1000, and 1500 µg; six HER2-positive in the 500 µg cohort)	FiH, prospective, open label	[59]

Another strategy to utilize the HER2-specific Affibody molecule for PET imaging is exemplified by the development of GE-226, in which the Affibody HER2 binder is labeled with ^18^F [60]. Kenny et al. presented the results of 20 breast cancer patients with locally advanced or metastatic disease imaged with this molecule at the 2022 Annual Meeting of the American Society of Clinical Oncology (ASCO). In the meeting abstract, the authors report on a significant difference in tracer uptake measured by SUVmean and SUVmax between biopsy-proven HER2-positive versus HER2-negative tumors. In 3 out of 20 patients, either intertumoral or intratumoral heterogeneity was observed, as depicted by varying degrees of tracer avidity. Of note, according to the authors, GE-226 imaging was able to distinguish lymphadenopathy due to sarcoidosis from malignant lesions in one patient and was superior to [^18^F]FDG PET in this case [56]. 

Other ^18^F-labeled HER2-Affibody ligands derived from ABY-025 coupled to two different chelators (NOTA and RESCA) were tested in early clinical trials [57]. In a direct comparison of Affibody-imaging compounds containing either the chelator NOTA or RESCA in five patients, the authors concluded that the RESCA-containing construct was superior. This molecule showed reduced kidney uptake and retention, as well as higher contrast images, and performed better in the detection of metastases [57]. Further studies with this compound are warranted to elucidate its full potential.

Lastly, two studies reported on the use of the HER2 Affibody variant [^68^Ga]Ga-NOTA-MAL-Cys-MZHER2:342. The first study demonstrated the translatability from preclinical to clinical application and the feasibility of imaging with this tracer in two breast cancer patients [53]. The second study highlighted various scenarios in which HER2 imaging can be of clinical utility in the assessment of breast cancer patients. Twenty-four patients with biopsy-/IHC-confirmed breast cancer participated in the trial. According to the authors of the study, treating physicians requested HER2 imaging for the following reasons: (1) to differentiate between metastases of multiple primary tumors with one confirmed primary HER2-positive breast cancer and additional primary cancers that could be breast cancer with no or low HER2 expression or other primary malignancies; (2) to assess the HER2 status of lesions not accessible to biopsy or repeat biopsy; and (3) to assess the heterogeneity of patients’ inter- and intratumoral HER2 expression [55]. Intra-patient tracer uptake heterogeneity was observed, and inter-patient tumor tracer uptake varied greatly (up to an 11-fold difference in SUVmax). Nonetheless, when compared to HER2 status detected on biopsies by IHC/ISH, the Affibody tracer demonstrated high sensitivity and specificity. Twenty-two out of 24 HER2-positive biopsied tumor lesions were also positive by HER2-Affibody-PET imaging. Tumor uptake with the ^68^Ga-labeled HER2-binding Affibody was considered positive in 16 patients, negative in 7 patients, and equivocal in 1 patient. Importantly, tumors from five patients previously classified as HER2-negative were reclassified as HER2-positive after imaging. Consequently, these patients showed an overall response rate of 60% after receiving anti-HER2 therapy due to imaging results. Conversely, two patients were reclassified as HER2-negative after imaging and received non-HER2 targeting therapy, resulting in an objective response in one of them. These results highlight how HER2-binding Affibody imaging can support personalized treatment decisions as well as overcome the shortcomings of biopsy-based HER2 assessment.

## 5. Clinical Experience of HER2-Affibody PET Imaging in HER2-Low Breast Cancer Patients

**Figure 3:**
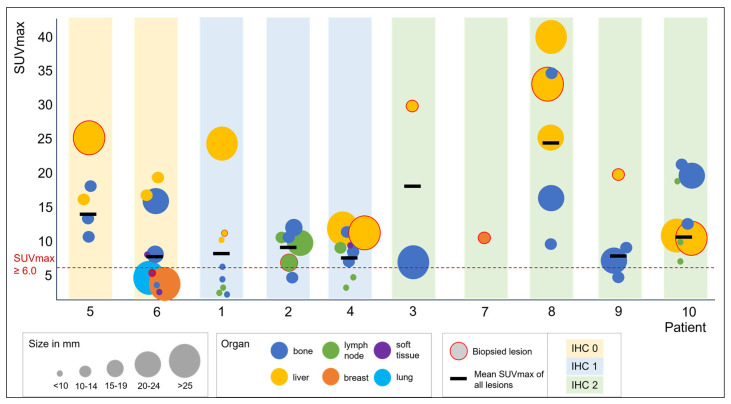
Results of [^68^Ga]Ga-ABY-025 PET/CT in 10 HER2-low breast cancer patients demonstrating disease heterogeneity. Bubbles represent standardized uptake values in single detected metastases; the different colors of the bubbles depict the location of the metastases in different organs, the relative size of the bubbles correlates to the actual size of the metastases, as explained in the figure legend. Per patient, the SUVmax of the lesion biopsied after HER2-PET imaging is indicated by a red circle around the colored bubble indicating the organ location of the biopsy. The colored bars (yellow, blue, and green) visualize the HER2 status of patients as assessed by biopsy prior to enrollment in the study. The mean SUVmax of all lesions is indicated by the thick black line. The SUVmax values of a maximum of three metastases per organ are shown. The data described in this figure was originally published in JNM by Altena et al. [58].

A first proof-of-concept trial utilizing [^68^Ga]Ga-ABY-025 PET/CT, including 10 previously biopsy-verified HER2-low breast cancer patients, was conducted as part of the NCT05619016 basket imaging trial for patients with HER2-expressing tumors. Results from this pilot cohort demonstrate that imaging of HER2-low patients with [^68^Ga]Ga-ABY-025 PET/CT is both safe and feasible [58]. All patients in this cohort showed lesions with [^68^Ga]Ga-ABY-025 uptake higher than background (Figure 3). After the ABY-025-PET investigation, the HER2-low status was confirmed in 8 of 10 patients by imaging-guided biopsy and subsequent IHC/ISH analysis. Two patients had biopsy results of HER2 0 in tumor lesions, one in a cutaneous lesion with low SUVmax and one in a liver metastasis with high SUVmax (24.9) but a ‘cold’ core due to central necrosis. Furthermore, substantial heterogeneity in tracer uptake was observed between different lesions within the same patient (Figure 3). The trial is planned to continue with the recruitment of new cohorts to validate [^68^Ga]Ga-ABY-025 PET/CT as a diagnostic tool for selecting patients for HER2-targeted therapy. The new cohorts will include the HER2-low patient group as well as patients with other tumor types with reported HER2-low expression and HER2-amplification.

## 6. SPECT Alternative for HER2-Affibody Imaging

**Figure 4:**
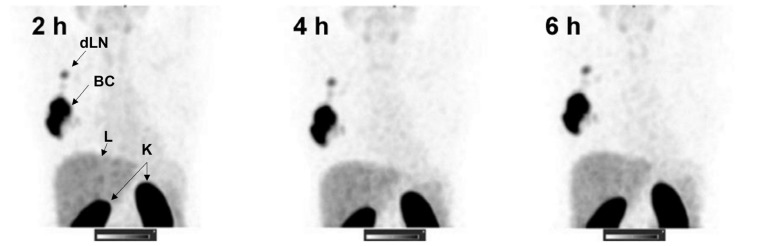
Representative SPECT images (MIP) at 2, 4, and 6 h after injection of [^99m^Tc]Tc-ZHER2:4107 (Patient 16) visualizing the primary breast cancer (BC) and tumor-draining lymph node (dLN). Kidney (K) and liver (L) are the normal organs with the highest tracer uptake. The images taken at 2, 4, and 6 h after tracer injection (pi) demonstrate that imaging is possible as early as 2 h pi and that tracer uptake in the tumor is constant from 2 to 6 h. In the pictures, a reduction in tracer uptake can be seen in the area of the noninvolved breast, stomach, and lungs by 6 h after injection. Imaging was performed after injection of the optimal defined peptide mass of 1000 μg [^99m^Tc]Tc-ZHER2:4107 that showed the best discrimination between HER2-positive and HER2-negative tumor lesions. Linear SUV scale from 0 to 15. Image adapted from [63]. Phase I clinical evaluation of ^99m^Tc-labeled Affibody molecule for imaging HER2 expression in breast cancer. Image adapted from a study published in Theranostics by Bragina et al. [63].

PET imaging is mostly available in highly developed countries and may not be available in countries with a less developed healthcare system [61,62]. Therefore, Bragina et al. studied the use of a modified Affibody tracer for SPECT/CT imaging in 31 primary breast cancer patients [63]. The applied tracer utilized the amino acid sequence GGGC instead of DOTA as a chelator for technetium-99m. ^99m^Tc is more easily available because of production in generators and is less expensive than ^111^In. It also provides lower absorbed doses and better spatial resolution than ^111^In. The short half-life of ^99m^Tc makes it suitable for imaging after only 2–4 h when coupled to a small peptide such as an Affibody molecule. To define the optimal injected peptide mass, three different mass doses of the Affibody tracer, i.e., 500, 1000, and 1500 μg, were tested in three treatment groups. A minimum of five HER2-positive and five HER2-negative patients were enrolled in each group. Patients received a radioactive dose of 451 ± 71 MBq [^99m^Tc]Tc-ZHER2:4107. SPECT/CT imaging was performed at several timepoints up to 24 h post tracer injection, and planar imaging was conducted at 2, 4, and 6 h after injection. This study defined 1000 μg of injected peptide mass as the superior dose for discriminating between HER2-positive and negative tumor lesions at an optimal imaging time point of 2 h (see representative images in Figure 4). 

All known lymph node metastases could be visualized, and significantly higher uptake in lymph node metastases in patients with HER2-positive lesions versus patients with HER2-low/negative lesions was observed. Further, all liver metastases could be visualized, with some liver metastases exceeding the uptake of tracer compared to primary tumor lesions. Kidneys, adrenal glands, and liver were the organs with the highest [^99m^Tc]Tc-ZHER2:4107 uptake, followed by breast and lung. Different protein mass doses did not have a major influence on healthy organ uptake of the tracer. 

The utility of Affibody-SPECT imaging for HER2-expressing cancers is further supported by a report on 30 breast cancer patients imaged with a [^99m^Tc]Tc-labeled HER2 Affibody [54]. In this study, 9 out of 30 patients had to be reclassified based on imaging results as either HER2-positive (six patients) or HER2-negative (three patients). HER2-Affibody SPECT imaging had an overall sensitivity of 80% and specificity of 60%, which increased to 100% sensitivity when a minimum tumor diameter threshold of 12 mm was applied. 

These studies, together with the early trial of [^111^In]In-ABY-025 in breast cancer patients [49], highlight the potential of SPECT/CT imaging for HER2 with Affibody-tracers, which may be a more feasible alternative to PET imaging, especially in low-income countries.

## 7. Affibody-Based HER2 Imaging Outside Breast Cancer

HER2 is not only overexpressed in breast cancer but, to varying degrees, also in other cancer types [4]. Most prominently in gastric and gastroesophageal cancer, overexpression rates between 9% and 35% have been reported [64], and HER2-targeted therapies are implemented as part of first-line (trastuzumab) and second-line (trastuzumab deruxtecan) treatment regimens [65]. Other cancer types with known HER2 expression include colorectal cancer, ovarian cancer, bladder cancer, and pancreatic cancer, and clinical trials with HER2-targeted compounds are expected to evaluate the targetability of HER2 alterations independent of primary indication in a tissue-agnostic way [66,67,68]. Notably, the DESTINY-PanTumor-02 trial is currently assessing the efficacy of trastuzumab deruxtecan in HER2-expressing cancers previously not indicated for Enhertu therapy (NCT04482309). The primary results of this Phase 2 trial show promising response rates in several cancer types, including endometrial, cervical, ovarian, and bladder cancer [69,70]. 

Similar to breast cancer, heterogeneity in HER2 expression has been reported in gastric cancer [71,72] and will be an emerging question in many other solid cancer indications with upcoming approvals of HER2-targeted agents. A study by Zhou and colleagues has indicated the utility of Affibody HER2-imaging in gastric cancer [73], where repeat biopsies to detect changes in HER2 expression may be particularly challenging due to the location of the lesion. Thirty-four patients with advanced gastric cancer were analyzed (23 HER2-positive, 11 HER2-negative primary lesions). All HER2-positive patients had lesions detectable by the Affibody ligand (^68^Ga-NOTA-MAL-MZHER2), and tracer uptake was three times higher in the HER2-positive patient group compared to the HER2-negative group. Maximum uptake in HER2-positive regions was highly variable and dependent on the target organ. Bone and liver metastases showed the highest tracer avidity, followed by lymph node metastases, and finally metastases at other sites. Notably, HER2-negative patients showed no site-dependent difference in tracer uptake. A case report on [^68^Ga]Ga-HER2-Affibody imaging of a 40-year-old woman with advanced metastatic gastric cancer published by the same authors was consistent with these results [74] and highlighted the superior ability of Affibody imaging compared to [^18^F]FDG PET in the detection of metastases in this patient. The authors concluded that imaging with the [^68^Ga]Ga-HER2-Affibody tracer enables a whole-body view of tumor load and HER2 expression and can be an adjunct diagnostic to guide treatment decisions in advanced gastric cancer.

The ongoing clinical trial NCT05619016 described above in the context of HER2-low breast cancer imaging also includes a cohort of patients with gastroesophageal cancer with known and unknown HER2-status for imaging with [^68^Ga]Ga-ABY-025 PET/CT. This trial will both help increase the understanding of HER2-expression and heterogeneity in this indication as well as validate Affibody HER2-PET imaging as a diagnostic tool in gastric cancer. Affibody-based imaging trials for HER2 are summarize Table 5.

**Table 5:** Affibody-based HER2 molecular imaging clinical trials in gastric cancer.

Affibody Ligand	HER2 Status	Radioisotope (PET/SPECT)	Number of Patients	Reference
NOTA-Mal-Cys-MZHer342	pos/neg	^68^Ga (PET)	34	Zhou EJNMMI 2021 [73]
NOTA-Mal-Cys-MZHer342	pos	^68^Ga (PET)	1	Zhou Clin Nucl Med 2020 [74]
ABY-025	all	^68^Ga (PET)	TBD	NCT05619016 (ongoing)

## 8. Future Directions

Molecular imaging for HER2 holds great promise for improving therapeutic decision-making and enabling truly personalized medicine through precision imaging. Due to the limitations of the current reference standard for identification of patients with HER2-expressing cancers based on biopsies, several other potential diagnostics are currently under clinical evaluation. Apart from molecular imaging, these comprise the analysis of liquid biopsies, where HER2 amplification or expression status is detected in circulating tumor DNA (ctDNA) or on circulating tumor cells [75]. Even though the first results of implementing ctDNA into patient selection for HER2-targeted therapy are encouraging, further work needs to be carried out to validate the clinical utility of such approaches [76]. A major drawback of utilizing liquid biopsies for patient stratification is that although they provide fast, minimally invasive, and cost-effective diagnostic insights, they will not visualize disease localization, tumor heterogeneity, or the extent of HER2-expression within patients and are, from this aspect, inferior to molecular imaging approaches. Another key advantage of molecular imaging of cancer targets is its potential application in a theranostic context [77]. In this context, imaging tracers to define the extent of treatable tumors within a patient before radiotherapeutic intervention will be of great advantage. Affibody imaging tracers may therefore find application in theranostic pairs consisting of an Affibody-imaging agent followed by targeted molecular radiotherapy. The concept of such an Affibody-based radiotherapy has been explored preclinically with promising results [78]. The translation of this approach into the clinical development of an HER2-targeted radioligand would highly benefit from the HER2-Affibody imaging technology.

## 9. Conclusions

Based on published data from more than 220 patients, Affibody-based HER2 molecular imaging appears safe and feasible. Considering the possibility of visualizing HER2 expression across the entire body, Affibody-HER2 PET or SPECT imaging can become an established diagnostic entity, overcoming the limitations of the current diagnostic standard based on biopsy analysis. Molecular imaging for HER2 could be an alternative when biopsies are not feasible or cannot be performed safely, or an adjunct or substitute for biopsies when biopsy results are deemed inconclusive or insufficient. This is particularly relevant considering that HER2 heterogeneity cannot be detected by single biopsies. The totality of data available for ABY-025 underlines its potential to assess HER2 receptor status safely and accurately—in breast cancer patients and beyond. Knowing the whole-body status of HER2 expression through molecular imaging as a diagnostic tool will greatly help to meaningfully guide the use of HER2-targeted therapies on an individual patient basis.

## Figures and Tables

**Table 1:** FDA-approved HER2-targeted therapeutics.

Name	Drug Type	Indication
trastuzumab (Herceptin^®^)	mAB	HER2+ BC (adjuvant, metastatic); HER2+ GC (metastatic)
pertuzumab (Perjeta^®^)	mAB	HER2+ BC (metastatic, locally advanced, inflammatory, or early stage)
ado-trastuzumab emtansine (Kadcyla^®^)	ADC	HER2+ BC (metastatic or early stage)
trastuzumab deruxtecan (Enhertu^®^)	ADC	HER2+ BC (unresectable or metastatic); HER2-mutant NSCLC; HER2+ GC and GEJ carcinoma (locally advanced or metastatic); HER2-low BC (metastatic)
margetuximab (Margenza^®^)	mAB	metastatic HER2+ BC
tucatinib (TUKYSA^®^)	TKI	HER2+ BC (locally advanced or metastatic)
lapatinib (Tykerb^®^)	TKI	HER2+ BC (advanced or metastatic; HR+ metastatic)
neratinib (NERLYNX^®^)	TKI	HER2+ BC (early stage or metastatic)

Abbreviations: ADC: antibody–drug conjugate; mAB: monoclonal antibody; TKI: tyrosine kinase inhibitor (small molecule); BC: breast cancer; GC: gastric cancer; HR: hormone receptor; NSCLC: non-small cell lung cancer; GEJ: gastroesophageal junction.

**Table 2:** Key advantages and limitations of PET and SPECT images.

Aspect	PET	SPECT
Cost and availability	Expensive equipment, limited availability	Less expensive, more common, and widely accessible
Image Quality	High resolution, range of clinical devices 2–6 mm	Lower resolution, range of clinical devices: 7–15 mm
Acquisition time	Short—dynamic imaging/kinetic modeling efficient	Long—dynamic imaging/kinetic modeling impractical
Quantification	Provides absolute quantification, excellent quantification	Somewhat lower quantification accuracy compared to PET

**Table 3:** Selected FDA or EMA approved PET and SPECT imaging tracers for oncology.

Name	Approval	Active Ingredient(s)	Indication
DetectNET^TM^	FDA	^64^Cu dotatate	SSTR+ neuroendocrine tumors
NETSPOT^®^	FDA	^68^Ga dotatate	SSTR+ neuroendocrine tumors
SomaKit TOC^®^	EMA	edotreotide (kit for radiolabeling with gallium-68)	SSTR+ neuroendocrine tumors
Octreoscan^TM^	FDA and EU national approvals	pentetreotide (kit for radiolabeling with indium-111)	SSTR+ neuroendocrine tumors
LYMPHOSEEK^®^	FDA and EMA	tilmanocept (kit for radiolabeling with technetium-99m)	Guiding sentinel lymph node biopsy in cancer patients; locating tumor-draining lymph nodes in adults and children.
LOCAMETZ^®^	FDA and EMA	gozetotide (kit for radiolabeling with gallium-68)	PSMA+ prostate cancer
Illuccix^®^	FDA	gozetotide (kit for radiolabeling with gallium-68)	PSMA+ prostate cancer
PYLARIFY^®^/PYLCLARY^®^	FDA and EMA	piflufolastat F-18	PSMA+ prostate cancer
Cerianna^TM^ *	FDA	fluoroestradiol F-18	ER+ breast cancer

Abbreviations: EMA: European Medicines Agency; ER: estrogen receptor; FDA: Food and Drug Administration; SSTR: somatostatin receptor; PSMA: prostate-specific membrane antigen; * also known as ESTROTEP in Europe.
